# The origin and course of the infrapatellar branch of the saphenous nerve: An anatomical study

**DOI:** 10.1016/j.jpra.2022.08.006

**Published:** 2022-09-05

**Authors:** David D. Krijgh, Emile B. List, Teun Teunis, Ronald L.A.W. Bleys, J. Henk Coert

**Affiliations:** a Department of Plastic and Reconstructive Surgery, University Medical Center Utrecht, The Netherlands; b Department of Orthopaedic Surgery, University Pittsburgh Medical Center, University of Pittsburgh, Pittsburgh, Pennsylvania; c Department of Anatomy, University Medical Center Utrecht, The Netherlands

**Keywords:** Infrapatellar branch, Saphenous nerve, Neuropathic pain, Diagnostic nerve block, Denervation

## Abstract

Nerve injury of the saphenous nerve or infrapatellar branch seems to be a frequent complication following knee surgery or trauma. Denervation results vary, and in some cases, no pain relief is achieved. This might be due to anatomic variation. The purpose of this anatomical study is to identify the variation in the course of the infrapatellar branch and saphenous nerve.

We dissected 18 cadavers from adult donors. Medial to the knee, the saphenous nerve and infrapatellar branch were identified and followed proximally to the point where the infrapatellar branch branched from the saphenous nerve. The location where the infrapatellar branch came off from the saphenous nerve relative to the knee joint and where it passed the knee joint were measured.

A total of 23 infrapatellar branches were found. We identified 10 branches between 0–10 cm proximal to the knee joint, 3 branches at 10–20 cm, and 9 branches at >20 cm. Between the patella and semitendinosus tendon, the knee joint was crossed by 5 branches in the anterior, 15 in the middle, and 2 in the posterior one-third.

The origin of the infrapatellar branch and the location at which it passes the knee are highly variable. This, in addition to people having multiple branches, might explain why denervation is frequently unsuccessful. Based on the anatomical findings, we propose a more proximal diagnostic nerve block to help differentiate between a distal-middle or proximal origin of the infrapatellar branch. Appropriate placement of the nerve block might help identify people who benefit from denervation.

## Introduction

The saphenous nerve is the largest sensory branch of the femoral nerve and provides cutaneous innervation to the medial part of the lower leg and foot.^1^ According to most descriptions, the infrapatellar branch separates from the saphenous nerve proximal to the medial femoral condyle as it innervates the skin over the anterior and medial region of the knee and inferior to the patella.^2^ Nerve injury of the saphenous nerve or infrapatellar branch is a frequent complication following knee surgery or trauma, with an incidence ranging from 21% to 69% depending on the type of surgery or trauma.^3^ Nerve injury can lead to neuropathic pain and can lower the quality of life significantly.^4^ The pain is described as sharp and burning, other symptoms include hyperalgesia, hypoesthesia, dysesthesia, and allodynia.^5^

In case non-operative therapies do not result in sufficient pain relief, surgical denervation can be considered.^6^ Selective denervation of the affected nerve can achieve long-term relief and improvement in patients’ quality of life.^4^^,^^7^ However, in our experience and that of others, the results vary, and in some cases, no pain relief is achieved.^8^ We hypothesize that this could be explained by anatomic variation. For adequate treatment, exact knowledge of the course of the saphenous nerve is required. We use nerve blocks to identify patients who might benefit from denervation. Knowing the exact location of the nerve through anatomic landmarks is essential for placing an adequate nerve block. Knowledge of anatomic variations is a prerequisite for a potentially successful denervation procedure. In addition, if we can reliably locate the infrapatellar branch, we may avoid the denervation of the complete saphenous nerve. A better understanding of the course of the infrapatellar branch and the saphenous nerve may also reduce iatrogenic injuries.

The purpose of this anatomical study is to identify the course of the saphenous nerve and the infrapatellar branch. Specifically, we aimed to answer the following questions: (1) where does the infrapatellar branch come off the saphenous nerve relative to the knee joint? (2) where do the infrapatellar and saphenous nerves cross the knee?

## Methods

### Study population

The lower limbs of 18 adult human cadavers were used, nine limbs were fresh-frozen, and nine were embalmed using 3% formaldehyde. These specimens were derived from bodies that entered the Department of Anatomy of the University Medical Center Utrecht through a donation program. Written informed consent was obtained from these individuals while alive, allowing their entire bodies to be used for educational and research purposes. One limb had a scar from a saphenous vein stripping procedure, the other limbs showed no macroscopic signs of disease or scarring. The age and sex of the cadavers are unknown due to anonymization.

### Approach to dissection

Dissections were performed by three authors (DK, EL, and TT) and supervised by the senior author. All limbs were dissected with the knee in an extended position. The dissection generally proceeded as follows: (1) to mark the medial part of the knee joint we inserted two Kirschner wires; one ventrally in the knee joint at the medial edge of the patellar apex; and one through the semitendinosus tendon into the posterior part of the knee joint. For practical reasons, we defined the knee joint as the line between the two Kirschner wires. (2) Medial to the knee, the saphenous nerve, and the infrapatellar branch were identified and followed proximally to the point where the infrapatellar branch branched from the saphenous nerve; (3) the location where the infrapatellar branch came off from the saphenous nerve relative to the knee joint and the point where it passed the knee joint, respective to the Kirschner wires, were measured.

### Measured variables

**Figure 1:**
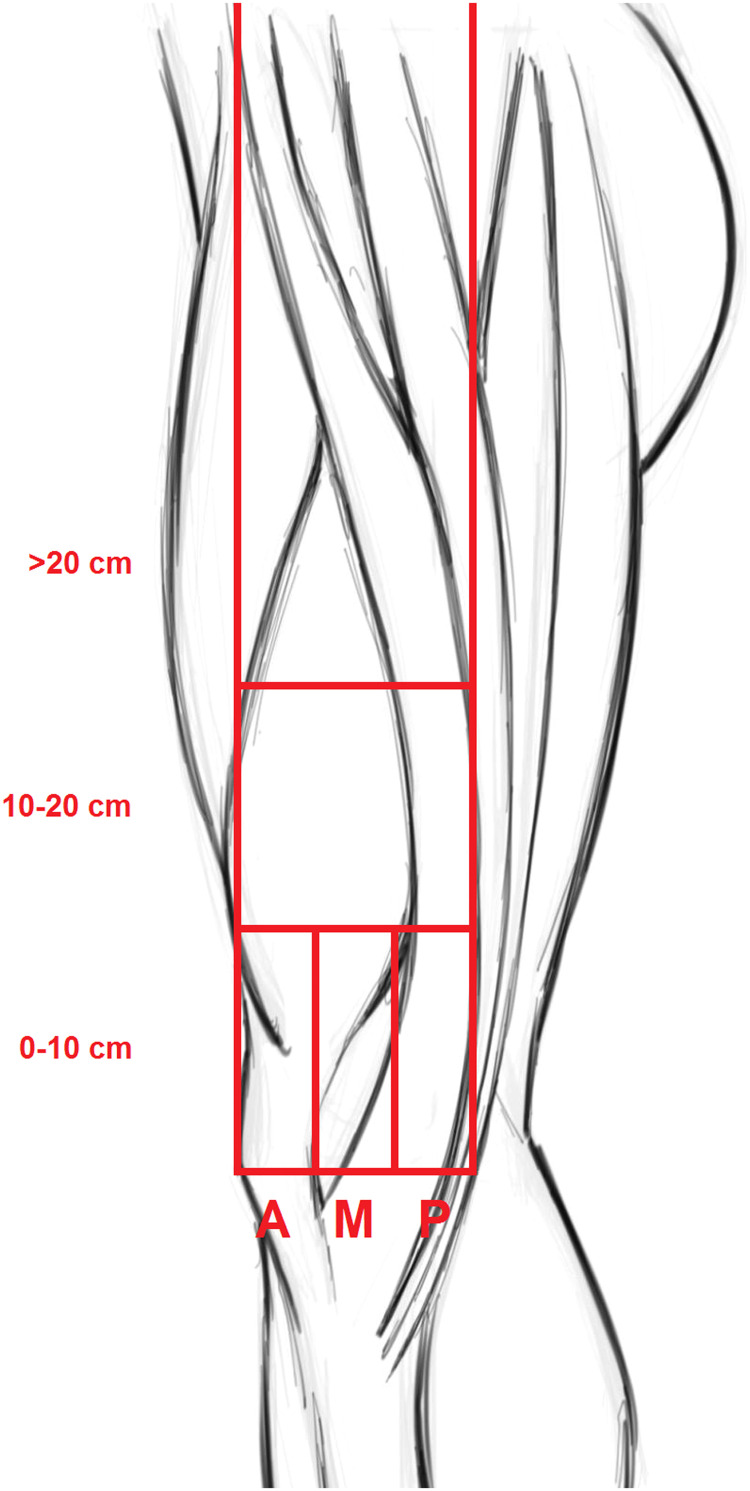
Illustrative horizontal and vertical segments. The horizontal and vertical lines used to illustrate the course of the nerves and the location of the branching pattern are shown. The infrapatellar branches are grouped based on distance of branching off from the saphenous nerve relative to the knee joint: distal (0–10 cm), middle (10–20 cm), proximal (>20 cm). The infrapatellar branches and saphenous nerves are grouped by segment in which the knee joint is passed: anterior (A), middle (M), and posterior (P) third.

We measured the following parameters: (1) vertical distance from the location of the infrapatellar branch coming off the saphenous nerve to the knee joint. Five of the legs were cut distal to the separation of the infrapatellar branch of the saphenous nerve, and the distance could not be determined. For these legs, we measured the length between the knee joint and the beginning of the infrapatellar branches at the point where the leg was cut; (2) mean horizontal distance from the medial border of the patellar apex to the infrapatellar branch; (3) mean horizontal distance from the medial border of the patellar apex to the saphenous nerve. The horizontal and vertical segments used to illustrate the course of the nerves and the location of the branching pattern are shown in Figure 1. The mean horizontal distance from the medial border of the patellar apex to the semitendinosus tendons, the infrapatellar branches, and the saphenous nerve is presented in Table 3. Photographs of each dissection were taken with the last generation iPhone (Apple Inc., Cupertino, California, USA).

### Number of branches

In the 18 dissected limbs, a total of 23 infrapatellar branches were found. At the level of the knee joint, one dissected limb had an additional infrapatellar branch, and two limbs had two additional branches. In one limb, only the proximal segment of the infrapatellar branch was identified because this person had undergone a saphenous vein stripping procedure.

### Statistical analysis

We identified 3 groups: (1) distal branching pattern, up to 10 cm proximal of the knee joint; (2) middle branching pattern, between 10 and 20 cm of the knee joint; (3) proximal branching pattern, more than 20 cm proximal to the knee joint. We report the distance between the knee joint and the infrapatellar branch branching point in mean distance within the proximal, middle, and distal groups. Four legs were cut close, but just distal, to the groin, after the separation of the infrapatellar branch. We grouped them into the proximal group, but we excluded them from the analysis of mean difference, since we could not determine a reliable branch point. One of the legs was cut 18 cm proximal to the knee joint. We could not reliably determine if this was a middle or proximal branch and therefore excluded this leg from the analysis. At the level of the knee joint, we divided the horizontal distance between the medial border of the patellar apex and the semitendinosus tendon in thirds: anterior, middle, and posterior. We report the distance between the medial border of the patellar apex to the infrapatellar branch and the saphenous nerve in mean distance in the anterior, middle, and posterior third.

## Results

### Where does the infrapatellar nerve come off the saphenous nerve?

Out of a total of 23 infrapatellar branches, the “distal branch” group included 10 branches that came off from the saphenous nerve between 0–10 cm proximal to the knee joint. In the “middle branch” group (10–20 cm proximal to the knee), three infrapatellar branches were identified. Nine branches were identified in the “proximal branch” group (infrapatellar nerves branching of >20 cm proximal to the knee joint). One infrapatellar branch could not be categorized because this specific leg was cut 18 cm proximal to the knee, and at this point, the infrapatellar branch was already separated from the saphenous nerve. (Table 1).

**Table 1:** Distance of branching off. The infrapatellar branches are grouped based on distance of branching off from the saphenous nerve relative to the knee joint.

Group	Number	Mean (cm)
Distal (0–10 cm)	10	4.6
Middle (10–20 cm)	3	13.3
Proximal (> 20 cm)	9	29.8
Not grouped	1	N/A

### Where do the infrapatellar branches and saphenous nerves cross the knee?

Five infrapatellar branches crossed the joint in the anterior one-third of the line between the two Kirschner wires used to mark the knee joint, 15 branches in the middle one-third, and two in the posterior one-third (Table 2).

**Table 2:** Position of infrapatellar branches (IPB) and saphenous nerves (SN) crossing the knee joint. An imaginary line between the medial patella and semitendinosus tendon is drawn and split in three. The infrapatellar branches and saphenous nerves are grouped by segment in which the knee joint is passed. The means represent the average distances between the medial border of the patellar apex and the infrapatellar branches or saphenous nerves.

Group	IPB (N)	IPB mean (mm)	SN (N)	SN mean (mm)
Anterior one-third	5	34	0	N/A
Middle one-third	15	70.7	5	73
Posterior one-third	2	110	13	111.5
Not grouped	1	N/A	0	N/A
Total	23	65.9	18	100.8

A total of 13 of the 18 saphenous nerves crossed the knee joint in the posterior one-third and five in the middle one-third of this line (Table 3).

**Table 3:** Mean horizontal distances at the level of the knee joint from the medial border of the patellar apex to the infrapatellar branches, the saphenous nerves, and the semitendinosus tendons.

Horizontal distance	Mean (mm)	SD (mm)	Range (mm)	Number
Medial patella - infrapatellar branch	65.9	26.9	10-115	22
Medial patella - saphenous nerve	100.8	26.5	45-150	18
Medial patella - semitendinosus tendon	138.3	21.8	110-190	18

## Discussion

The saphenous nerve and the infrapatellar branch are prone to nerve damage after surgery or trauma, specifically due to anterior cruciate ligament reconstructions and open tibial fracture, possibly resulting in debilitating neuropathic pain.^3^^,^^4^ We noticed that the courses of the saphenous nerve and the infrapatellar branch are inconsistent. A better understanding of the origin of the infrapatellar branch can be helpful with patient selection for nerve blocks and surgical denervation. The results demonstrated that the branching off point of the infrapatellar branch was highly variable as was the location where it passed the knee joint. The course of the saphenous nerve is more consistent, and the nerve passes the knee joint near the semitendinosus tendon. We propose a more proximal diagnostic ultrasound-guided nerve block to better localize the origin of the infrapatellar branch. Future clinical studies will be necessary to validate this diagnostic nerve block and determine if it improves patient-reported outcome after denervation.

### Limitations

This anatomical study has several limitations. First, the availability of donor limbs for this study was limited; as a result, rare anatomical variations may have been missed. However, we already identified a high variability in our sample and do not suspect that this will change much by increasing the sample size. A larger sample size might increase precision within the different categories identified. Secondly, five legs were cut distal to the groin, after the separation of the infrapatellar branch. Therefore, one branch could not be grouped, and the other branches were grouped in the proximal group (>20 cm). Our sample size is too small for multiple imputation – the ideal method to handle the missing date. Therefore, we decided to omit the 5 legs which were cut distal to the groin and in which no reliable point of branching could be determined from the mean distance analysis. Lastly, because several specimens were cut off distal to the groin, we could not account for total leg length. A total of 10 cm proximal to the knee joint is arbitrary and might have different implications in short compared to long legs. However, our proposed diagnostic nerve block does not change because of person-to-person variation.

### Where does the infrapatellar branch come off the saphenous nerve?

There seems to be no clear pattern in the location where the infrapatellar nerve branches off from the saphenous nerve. In our study, 10/23 infrapatellar branches branched off between 0–10 cm proximal to the knee joint, 3/23 between 10–20 cm, and 9/23 >20 cm. An anatomical study in 22 cadaveric limbs conducted by Vanamala et al. concluded that all infrapatellar separations were found in the distal or middle one-third of the adductor canal.^9^ These distal 2/3rds of the canal are located between 0–20 cm proximal to the knee joint. This differs from our study in which nine of the branches were bifurcated >20 cm proximal to the knee. However, both studies show that the location of the bifurcation differs, and anatomical variations are common. This complicates both diagnostic nerve blocks and surgical denervation. Instead of the diagnostic nerve block used in the current clinic, we propose a more proximal ultrasound-guided saphenous diagnostic nerve block at the border of the proximal and middle third of the leg. In case, this block is effective the infrapatellar branch, or branches, come off in the middle or distal segment; if the block is ineffective, then the branch, or branches, separate in the proximal third of the upper leg. After the diagnostic nerve block and ineffective non-operative therapy, the surgeon can offer an attempt at denervation. Proximally, we found that several infrapatellar branches came off the saphenous nerve posterior to the inguinal ligament, making it a difficult denervation. It is important to keep in mind that this proximal diagnostic nerve block does not account for multiple branches. We found two infrapatellar branches in 1/23 dissected limbs and three in 2/23 limbs. Walshaw et al. found a higher prevalence of multiple saphenous branches: two branches in 8/25 dissected specimens and three branches in 6/25.^10^ This suggests a high potential failure rate of denervation – this is something to counsel every patient considering denervation on. Future study should determine whether the proposed diagnostic nerve block really enhances the successful denervation.

### Where do the infrapatellar branch and saphenous nerves cross the knee?

In our study, the location of the infrapatellar branch crossing the knee joint was highly variable. At the level of the knee joint, only 2/23 infrapatellar branches crossed the joint in the posterior one-third. The mean horizontal distance from the medial border of the patella apex to the infrapatellar branch was 6.6 cm (range 1.0 to 11.5 cm). Ebraheim et al. examined the course of the infrapatellar branch in 28 limbs and found an average horizontal distance from the patellar apex to the branch of 6.7 cm with values ranging from 3.4 to 10.5 cm.^2^ Henry et al. measured the same distance in 200 cadaveric limbs and found a mean of 4.6 cm.^11^ Mochida et al. dissected 129 knees and found a mean of 4.4 cm with values ranging from 1.2 to 9.0 cm.^12^ These studies and ours confirm a highly variable course of the infrapatellar branch near the knee joint. Regarding the saphenous nerve, our results show that the course around the knee joint was less variable than the infrapatellar branch. Most of the nerves (13/18) crossed the imaginary horizontal line, between the patellar apex and the semitendinosus tendons, in the posterior third. The mean horizontal distance from the semitendinosus tendon to the saphenous nerve, at the level of the knee joint, was 3.8 cm (range 1.0 to 6.5 cm). Dunaway et al. evaluated 100 knee MRIs to evaluate the same parameter and found a shorter mean distance of 1.5 (range 0.5 to 3.0 cm).^13^ This difference might be explained by the different methods used (cadavers vs. MRI). These results do indicate that most of the saphenous nerves cross the knee joint in the posterior part, which is consistent with our findings. The anatomic variance of the infrapatellar branch, in particular, seems to make it unlikely that we can identify reliable anatomic safe zones. This explains the high iatrogenic injury rate. Surgeons should be careful during any procedure on the medial side of the knee. Also, we have learned from this that denervation at this level will be less successful due to the anatomical variation.

## Conclusion

The origin of the infrapatellar branch from the saphenous nerve is highly variable, as well as where the infrapatellar branch passes the knee joint. This high variability, and the fact that some people have multiple infrapatellar branches, may help explain why denervation of the infrapatellar branch or the saphenous nerve is frequently unsuccessful. Based on the anatomical findings presented, we propose a more proximal diagnostic ultrasound-guided nerve block to help differentiate between a distal and middle, or proximal origin of the infrapatellar branch. This diagnostic nerve block might improve the decision if surgical denervation is a potential treatment option.

## Ethics

These specimens were derived from bodies that had entered the Department of Anatomy of the University Medical Center Utrecht through a donation program. Written informed consent was obtained from these persons when they were alive.

## Funding

No funding was received.

## Declaration of Competing Interest

No author has any form of conflict of interest.
